# Ecotypic differences in the phenology of the tundra species *Eriophorum vaginatum* reflect sites of origin

**DOI:** 10.1002/ece3.3445

**Published:** 2017-10-19

**Authors:** Thomas C. Parker, Jianwu Tang, Mahalia B. Clark, Michael M. Moody, Ned Fetcher

**Affiliations:** ^1^ The Ecosystems Center Marine Biological Laboratory Woods Hole MA USA; ^2^ Biological Sciences University of Texas at El Paso El Paso TX USA; ^3^ Institute for Environmental Science and Sustainability Wilkes University Wilkes‐Barre PA USA; ^4^ Present address: Biological and Environmental Sciences Faculty of Natural Sciences University of Stirling Stirling UK

**Keywords:** Arctic tundra, common garden, ecotypes, *Eriophorum vaginatum*, growing season length, local adaptation, phenology, senescence

## Abstract

*Eriophorum vaginatum* is a tussock‐forming sedge that contributes significantly to the structure and primary productivity of moist acidic tussock tundra. Locally adapted populations (ecotypes) have been identified across the geographical distribution of *E. vaginatum*; however, little is known about how their growth and phenology differ over the course of a growing season. The growing season is short in the Arctic and therefore exerts a strong selection pressure on tundra species. This raises the hypothesis that the phenology of arctic species may be poorly adapted if the timing and length of the growing season change. Mature *E. vaginatum* tussocks from across a latitudinal gradient (65–70°N) were transplanted into a common garden at a central location (Toolik Lake, 68°38′N, 149°36′W) where half were warmed using open‐top chambers. Over two growing seasons (2015 and 2016), leaf length was measured weekly to track growth rates, timing of senescence, and biomass accumulation. Growth rates were similar across ecotypes and between years and were not affected by warming. However, southern populations accumulated significantly more biomass, largely because they started to senesce later. In 2016, peak biomass and senescence of most populations occurred later than in 2015, probably induced by colder weather at the beginning of the growing season in 2016, which caused a delayed start to growth. The finish was delayed as well. Differences in phenology between populations were largely retained between years, suggesting that the amount of time that these ecotypes grow has been selected by the length of the growing seasons at their respective home sites. As potential growing seasons lengthen, *E. vaginatum* may be unable to respond appropriately as a result of genetic control and may have reduced fitness in the rapidly warming Arctic tundra.

## 1 INTRODUCTION

Climate change may force species into decline and eventual extinction as local environmental conditions change and the geographical ranges of suitable growth conditions shift (Diffenbaugh & Field, 2013; Thomas et al., 2004). The climate of the Arctic is warming at a faster rate than the rest of the planet (Serreze & Barry, 2011) and may warm by up to 11°C by 2,100 should emissions of greenhouse gases follow their current trajectories (IPCC 2013). Several arctic plant species show strong ecotypic differentiation across broad geographical ranges due to local selection pressure; these may be slow to react to climate change especially if there is limited gene flow between populations (Bennington et al., 2012; Chapin & Chapin, 1981; McGraw & Antonovics, 1983; McGraw et al., 2015). In recent years, arctic plants have shown increases in growth and cover across many areas of the tundra (Elmendorf, Henry, Hollister, Bjork, Boulanger‐Lapointe, et al., 2012); however, the response is not consistent across all plant functional types (PFTs). This raises the question of whether climate change will offer opportunities to some PFTs while the response of others will be constrained by their recent evolutionary history.

*Eriophorum vaginatum* L. (Cyperaceae) has demonstrated ecotypic differentiation across environmental gradients in the tundra and boreal ecosystems of Alaska (Shaver, Fetcher, & Chapin, 1986). *E. vaginatum* is a tussock‐forming sedge and a foundation species (Ellison et al., 2005) of moist acidic Arctic tundra, which contributes up to 30% of annual primary productivity (Chapin & Shaver, 1985; Shaver, Bret‐Harte, & Jones, 2001) and provides the characteristic tussock structure to moist acidic tundra (Chapin, van Cleve, & Chapin, 1979). Populations of *E. vaginatum* have been shown to produce less biomass per tiller along a latitudinal gradient in Alaska from south to north (Shaver et al., 1986). When populations are transplanted into common gardens north or south of their site of origin, they continue to show differences in leaf biomass production 3 years after being moved (Fetcher & Shaver, 1990). After reciprocal transplanting, “home site advantage” is shown, whereby tussocks growing at the site of their origin have fitness advantages in photosynthetic rates and biomass per leaf (Souther, Fetcher, Fowler, Shaver, & McGraw, 2014), new tiller production (McGraw et al., 2015), and ultimately survival (Bennington et al., 2012).

Northern populations of *E. vaginatum* have particularly low productivity when transplanted south into a warmer environment (Fetcher & Shaver, 1990). On the other hand, shrubs in the genera *Betula, Salix,* and *Alnus* have been observed to increase in growth rates and cover across the circumpolar region, primarily in response to increasing summer temperatures and extended growing seasons (Elmendorf, Henry, Hollister, Bjork, Boulanger‐Lapointe, et al., 2012; Myers‐Smith et al., 2011). This is consistent with experiments which show that shrub growth in the low Arctic often responds more positively than sedges to warming using open‐top chambers (OTCs) (Elmendorf, Henry, Hollister, Bjork, Bjorkman, et al., 2012; Walker et al., 2006). If *E. vaginatum*‐dominated tundra were to shift to higher shrub coverage, ecosystem processes will shift with changes in productivity and carbon turnover (Cahoon, Sullivan, Shaver, Welker, & Post, 2012).

Phenology, the timing of plant processes in relation to environmental cues, is a key control on productivity (Cleland, Chuine, Menzel, Mooney, & Schwartz, 2007) and could be sensitive to climate change (Walther et al., 2002). The phenology of plants is known to respond to temperature and photoperiod (Richardson et al., 2012; Tang et al., 2016), but in high‐latitude ecosystems 24‐hr days persist late into the growing season, meaning that plants may need other cues to trigger senescence including changes in light quality including the red:far‐red light ratio (Nilsen, 1985) as well as temperature (Clapham, Ekberg, Eriksson, Norell, & Vince‐Prue, 2002; Clapham et al., 1998; Mølmann, Junttila, Johnsen, & Olsen, 2006). Studies of plant phenology from temperate and boreal regions using common garden experiments have demonstrated that plants from higher latitudes flower earlier (Olsson & Ågren, 2002; Weber & Schmid, 1998). However, the opposite has been shown in other study systems (Kalisz & Wardle, 1994). At present, ecotypic differentiation in plant phenology remains plausible but with limited evidence in the literature.

In arctic ecosystems, plant growth is constrained by a very short growing season (Euskirchen et al., 2006). Furthermore, the seasonal shifts from cold to warm and back again can occur extremely rapidly (Cherry et al., 2014). The length of the growing season at different sites could produce strong local selection for the timing of green‐up and senescence in arctic plants. Over recent decades, warmer temperatures and smaller winter snowpacks have caused a significant lengthening of the potential growing season in arctic ecosystems, with both earlier thaw and later freeze‐up (Euskirchen et al., 2006; Park et al., 2016). However, snow removal and addition experiments to manipulate the start of the growing season have shown that phenology can be shifted, resulting in earlier green‐up followed by earlier senescence (Rosa et al., 2015; Semenchuk et al., 2016). In both of the cited experiments, there was no net increase in growth period following the earlier onset of growth, suggesting that there may be a genetic basis for duration of seasonal growth (Rosa et al., 2015). These results raise the question whether locally adapted arctic plants have the capacity to respond to longer growing seasons or whether other factors associated with climate change (e.g., community change and stimulation of growth [Elmendorf, Henry, Hollister, Bjork, Bjorkman, et al., 2012]) are driving observed (Epstein et al., 2012) “greening” patterns.

Until now, growth rates and phenology of different ecotypes of *E. vaginatum* have not been documented in detail through a growing season. In this study, we measured growth rate of leaves, the date at which senescence started, and the date at which individuals reached their peak in greenness for six populations of *E. vaginatum* collected along a latitudinal gradient 4 years after transplanting into a common garden. Specifically, we addressed the following questions: (1) Are there ecotypic differences in growth rate and phenology that correspond with previously observed differences in biomass accumulation (Shaver et al., 1986)? We hypothesized that ecotypes from further south with warmer, longer growing seasons would grow faster and senesce later. (2) Can phenology and growth rates be influenced by experimental warming? Our second hypothesis was that these traits would remain unaffected by warming because they have been under strong selection pressure and have limited capacity to respond to environmental change.

## 2 MATERIALS AND METHODS

### 2.1 Site description and experimental design

A common garden with six different populations of the tussock‐forming sedge, *E. vaginatum*, taken from different latitudes in Alaska was established in moist acidic tussock tundra near Toolik Lake, Alaska (68°38′N, 149°36′W), during summer 2011. Vegetation at this site is dominated by tussocks of *E. vaginatum*, deciduous dwarf shrubs (*Betula nana* L. and *Salix* spp.) and evergreen shrubs (*Vaccinium vitis‐idaea* L., *Rhododendron tomentosum* Harmanja [previously *Ledum palustre*], *Cassiope tetragona* L.) along with an understorey of mosses. The garden consisted of four replicate plots of plants from the six different populations of *E. vaginatum* (three tussocks in each plot). Tussocks were taken from three sites south of the Brooks Range and three sites north of the Range. The southern sites were Eagle Creek (EC; 65°26′N, 145°31’W), No Name Creek (NN; 66°07′N, 150°10’W), and Coldfoot (CF; 67°15′N, 150°10’W) and the northern sites were Toolik Lake (TL; locally transplanted as a reference), Sagwon (SG; 69°25′N, 148°43’W), and Prudhoe Bay (PB; 70°20′N, 149°04’W). These six sites were used for previous long‐term studies (Bennington et al., 2012; Shaver et al., 1986; Souther et al., 2014). Tussocks were transplanted in July 2011 according to the protocol of McGraw et al. (2015). A serrated knife was used to sever the rhizomes from roots and soil at a tussock's base and remove it from the tundra. Tussocks from each site, including TL, were then placed in the vacant positions at the common garden where local tussocks had been removed. This method has a high success rate due to *E. vaginatum*'s deciduous rooting strategy; although roots are severed during transplanting, new roots grow in each subsequent year, restoring full root function (Fetcher & Shaver, 1990; McGraw et al., 2015). Plots were arranged in pairs of the same population, and the spatial arrangement of these pairs was random. One of each of the pairs was randomly selected to be passively warmed using an OTC. The OTC has a cone shape with a bottom diameter of 1.23 m and top diameter of 0.84 m and vertical height 0.7 m, as modified from the OTCs used by the International Tundra Experiment (ITEX) (Marion et al., 1997) by increasing the diameter and height (J. Tang, personal communication, 2011). The OTCs were placed on the selected plots in 2015 from 11 July until 28 August and in 2016 from 2 June until 28 August and caused a mean hourly air temperature increase of 1.16°C at 20 cm above the ground and an increase of 2.41°C when photosynthetically active radiation (PAR) was above 600 μmol m^−2^ s^−1^.

### 2.2 Leaf measurements

Leaf growth and senescence were monitored from 17 June until 21 August in 2015 and from 2 June until 26 August in 2016. One tiller from the northernmost tussock of each plot was selected haphazardly to monitor using tagging and measuring methods similar to those described in Shaver and Laundre (1997). In each case, a small zip tie was secured around the base of the tiller, so as to include all leaves with any visible green portions, but to exclude any previously senesced leaves from previous growth. The total leaf length and the length of the green portions were measured to the nearest 5 mm approximately once a week for each leaf in a tiller, from oldest to youngest.

### 2.3 Environmental datasets

Data for cumulative thawing degree‐days (TDD, defined as the sum of the mean daily temperatures over 0°C), air temperature, and PAR in 2015 and 2016 were acquired from the meteorological stations operated by the Toolik Field Station Environmental Data Center of the University of Alaska, Fairbanks (Environmental Data Center Team). Mean potential growing season length and mean annual TDD for each of the six sites of origin were extracted and calculated from the SNOTEL database (http://www.wcc.nrcs.usda.gov/snow/). Here, growing season length was defined as the number of consecutive days of daily temperatures at or above 0°C.

### 2.4 Data processing

The study population consisted of 24 tillers, four each from six populations. The senesced portions of leaves were fragile and sometimes broke off; as this occurred after leaves had reached their full length, the total length was corrected to match the last measurement of the unbroken leaf. Where lengths of single leaves were missing for a time point due to human error, they were replaced with the mean of the previous and following time points. Only leaves that were growing during the season of measurement were measured, thereby excluding leaves that were grown in the previous year and were senescing as well as leaves that had been initiated for the next year but were not elongating. Growth rate was determined for these leaves for both years over similar time periods from mid‐June until early August to permit comparisons between years. The date when senesced portions of actively growing leaves were first observed was identified as the onset of senescence for that tiller. In some cases, no senescence was observed and the last observation date in the dataset was used. We acknowledge that this may be an underestimate of the date of actual senescence as defined above. The date at which the maximum green length of leaf was observed on the whole tiller was also determined. Maximum green length for each tiller was taken to be the maximum value of the summed green leaf lengths of the whole tiller over the course of each growing season.

### 2.5 Statistical analysis

Linear mixed‐effects models fitted with restricted maximum likelihood (REML) were used to analyze the effect of population, warming, and year as fixed effects on the response variables growth rate, senescence date, maximum green date, and maximum green length. Mixed‐effects models were applied using the “nlme” package in R (Pinheiro, Bates, DebRoy, & Sarkar, 2017; R Development Core Team 2016). The residuals of each model were normally distributed. Plot identity within the experiment was identified as a random factor within the model in the analyses. SG individuals needed to be removed from the analysis of growth rate due to insufficient replication. The relationship of these response variables in either the growing season length or the TDD at the population's “home” site was analyzed using linear regression models. All analyses were carried out with R v3.3.3 (R Development Core Team 2016).

## 3 RESULTS

Air temperature and light levels differed substantially between 2015 and 2016 over the duration of the growing seasons (Table 1). In May, at the beginning of the growing season, mean daily temperature was 3.8°C warmer in 2015 than 2016, which averaged only 0.8°C (Table 1). June 2015 remained on average 2.4°C warmer than 2016. Through both of these months, daily PAR was similar (Table 1, Figure S1). Later in the growing season, the difference in temperature shifted and 2016 was warmer than 2015. The average temperature for July was 1.5°C warmer in 2016 than in 2015 and August averaged 3.6°C warmer in 2016. The late warmth in August 2016 coincided with higher mean light levels (261.3 μmol m^−2^ s^−1^ [Table 1, Figure S1]) than in August 2015 (178 μmol m^−2^ s^−1^ [Table 1, Figure S1]). Over the growing season, daily temperatures were marginally warmer in 2015 (7.1°C) than in 2016 (6.9°C).

**Table 1:** Mean daily temperature and photosynthetically active radiation (PAR) data at Toolik Lake Field Station over the growing season in 2015 and 2016. In 2015 and 2016, winter snowpack melted on day 137 and 138, respectively (17 and 18 May)

	Air T (°C)		Max air T (°C)		Min air T (°C)		PAR (mol m^−2^ s^−1^)	
	2015	2016	2015	2016	2015	2016	2015	2016
May	4.6	0.8	10.4	5.7	−1.4	−4.4	449.8	451.4
June	9.0	6.6	14.0	11.5	3.3	−0.4	438.9	458.7
July	10.1	11.6	14.8	16.4	3.4	5.4	416.0	441.8
August	4.9	8.5	8.6	13.2	1.4	3.5	178.0	261.3
Average	7.1	6.9	11.9	11.7	1.7	1.0	370.7	403.3

### 3.1 Maximum green leaf length

**Figure 1:**
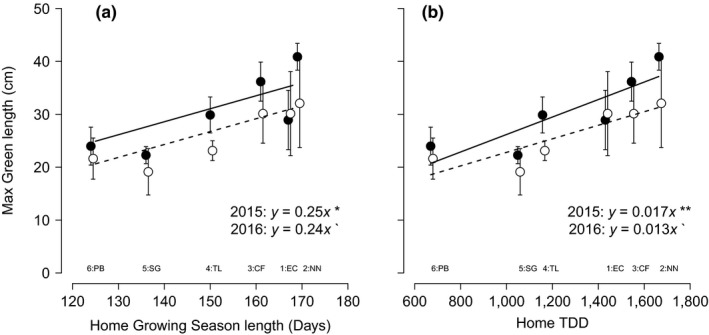
Maximum length of green leaves of six populations of *Eriophorum vaginatum* in the common garden in relation to (a) growing season length or (b) thawing degree‐days (TDD) of the site of origin of the population. Data from 2015 are represented by filled circles and a solid line, and data from 2016 are represented by open circles and a dotted line. Correlation coefficients and lines are displayed if *p *<* *.1, and the level of significance is indicated as follows: *p *>* *.1`, *p *>* *.5*, *p *>* *.01**. Error bars represent ± 1 standard error of the mean. Abbreviations of site of origin names are provided below to aid interpretation. The number at each site abbreviation relates to the relative latitude of each site (1–6 from south to north)

There were no significant differences between populations in the maximum green leaf length over the growing season, and there was no effect of passive warming (Table 2). There was a significant difference between years whereby leaves grew longer in 2015 than in 2016 (Table 2). In 2015, maximum green length was positively correlated with length of the growing season at the site of origin (Figure 1a). In 2016, this trend was also present but with more variation within the populations from longer growing seasons which resulted in a weaker relationship. The correlation coefficient was, however, very similar in both years (Figure 1a). This pattern was also apparent with regard to TDD where the relationship was stronger in 2015 (Figure 1b).

**Table 2:** Test statistics from linear mixed‐effects models analyzing the effect of population, warming, and study year on four variables: growth rate, senescence starting date, date at which green leaves are at their maximum and the maximum green leaf length. Significant results (*p *<* *.05) are in bold

Factor	Growth rate			Senescence starting date			Max green leaf date			Max green leaf length		
	*df*	*F*	*p*	*df*	*F*	*p*	*df*	*F*	*p*	*df*	*F*	*p*
Population	4,9	0.25	.903	**5,12**	**3.43**	**.037**	**5,12**	**3.50**	**.035**	5,12	1.29	.330
Warming	1,9	0.07	.801	1,12	0.00	.965	1,12	0.16	.699	1,12	0.04	.839
Year	1,9	0.55	.478	1,10	2.26	.163	**1,12**	**15.97**	**.002**	**1,12**	**8.29**	**.014**
Population × Warming	4,9	0.69	.619	5,12	0.10	.991	5,12	0.58	.712	5,12	0.14	.980
Population × Year	4,9	1.36	.320	5,10	2.28	.126	5,12	2.70	.074	5,12	0.96	.479
Warming × Year	1,9	1.38	.270	1,10	0.53	.482	1,12	0.23	.637	1,12	2.16	.167
Population × Warming × Year	4,9	0.86	.524	5,10	0.47	.793	5,12	1.88	.172	5,12	0.80	.571

### 3.2 Leaf growth rate

**Figure 2:**
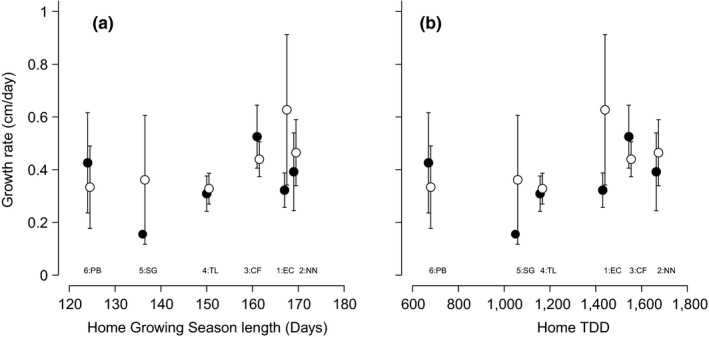
Growth rates of green leaves of six populations of *Eriophorum vaginatum* in the common garden in relation to (a) growing season length or (b) thawing degree‐days (TDD) of the site of origin of the population. Data from 2015 are represented by filled circles, and data from 2016 are represented by open circles. Correlation coefficients and lines are displayed if *p *<* *.1. Error bars represent ± 1 standard error of the mean. Abbreviations of site of origin names are provided below to aid interpretation. The number at each site abbreviation relates to the relative latitude of each site (1–6 from south to north)

There were no significant differences in growth rate of leaves between different populations and no effect of warming treatment. There were also no significant differences in growth rate between sampling years (Table 2). Growth rate was not significantly correlated with length of the growing season length or TDD of the home sites of the populations for either the sampling periods of 2015 or 2016 (Figure 2a,b).

### 3.3 Senescence start date

**Figure 3:**
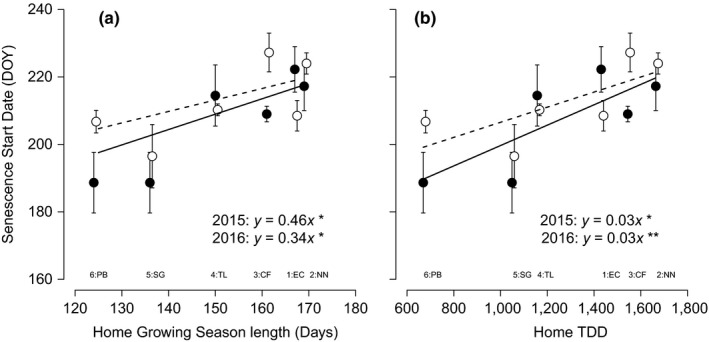
The day of the year at which senescence was first observed of six populations of *Eriophorum vaginatum* in the common garden in relation to (a) growing season length or (b) thawing degree‐days (TDD) of the site of origin of the population. Data from 2015 are represented by filled circles and a solid line, and data from 2016 are represented by open circles and a dotted line. Correlation coefficients and lines are displayed if *p *<* *.1, and the level of significance is indicated as follows: *p *>* *.5*, *p *>* *.01**. Error bars represent ± 1 standard error of the mean. Abbreviations of site of origin names are provided below to aid interpretation. The number at each site abbreviation relates to the relative latitude of each site (1–6 from south to north)

The date of start of senescence was significantly related (*p *=* *.037) to differences in populations of *E. vaginatum* in the common garden at Toolik Lake (Table 2). In 2015, plants from the southernmost ecotype, EC, were the last to senesce. As the site of origin was displaced further northwards, the date of senescence generally became earlier (Figure 3a). In 2016, the same trend was maintained, except that the CF population was the last to begin senescing (Figure 3a, Table 2). Plants from most populations started to senesce later in 2016 than in 2015, although this effect was not found to be statistically significant (*p *=* *.16). In both 2015 and 2016, there were significant positive relationships between the length of the growing season at the site of origin and the date of start of senescence of plants from the populations in the common garden experiment (Figure 3a). Although the relationship between these variables remained positive from 2015 to 2016, it was less pronounced as the correlation coefficient was reduced from 0.46 to 0.34 (Figure 3a). A higher intercept in the regression equation in 2016 indicates a general trend toward later senescence in 2016 than in 2015 (Figure 3a,b). The starting date of senescence was also significantly positively related to the TDD of the sites of origin in both 2015 and 2016 (Figure 3b).

### 3.4 Maximum green leaf length date

**Figure 4:**
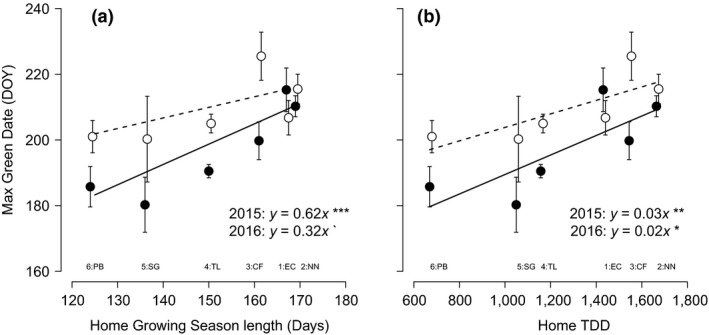
The day of the year at which green leaf length was at its maximum of six populations of *Eriophorum vaginatum* in the common garden in relation to (a) growing season length or (b) thawing degree‐days (TDD) of the site of origin of the population. Data from 2015 are represented by filled circles and a solid line, and data from 2016 are represented by open circles and a dotted line. Correlation coefficients and lines are displayed if *p *<* *.1, and the level of significance is indicated as follows: *p *>* *.1`, *p *>* *.5*, *p *>* *.01**, *p *>* *.001***. Error bars represent ± 1 standard error of the mean. Abbreviations of site of origin names are provided below to aid interpretation. The number at each site abbreviation relates to the relative latitude of each site (1–6 from south to north)

**Figure 5:**
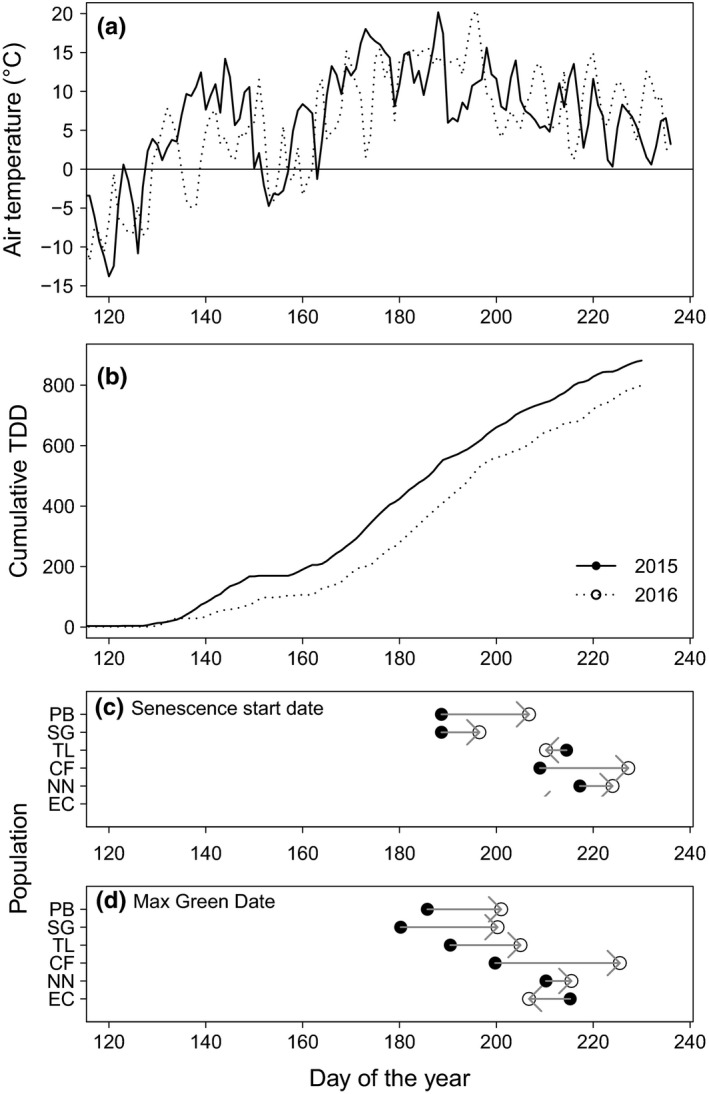
Air temperature (a) and cumulative thawing degree‐days (TDD) (b) in 2015 (solid line) and 2016 (dotted line) at Toolik Lake, AK compared with the mean senescence start date (c) and maximum green date (d) in either year (2015: closed circles, 2016: open circles) of different *Eriophorum vaginatum* populations at the same site. The arrows indicate the direction of change between 2015 and 2016 for each population

The date of maximum length of green leaves, which sums across all the leaves in the tiller in all stages of development and senescence, showed trends similar to the starting date of senescence (Figure 4a,b). The date of maximum green leaf length differed significantly between populations (*p *=* *.035) with the southern populations having longer growing seasons and reaching their peak later in the season. This date was significantly later across all populations in 2016 than in 2015 by 12 days (*p *=* *.002, Table 2). Every population except EC shifted to a later date (Figure 5). In 2015, the date at which green leaf length reached its maximum was closely correlated to the length of the growing season at the site of origin (*p *<* *.001, Figure 4a,b); in 2016, this correlation was still present but with a less steep slope (0.62 reduced to 0.32). As in the case of senescence date, the y intercept of the regression equation was higher than in 2015. Very similar patterns were observed in 2015 and 2016 between home site TDD and the date at which tillers reached their maximum green length (Figure 4b). Warming had no effect on either the start of senescence or the date at which maximum green length was reached on tillers (Table 2).

More TDD were accumulated in 2015 than in 2016. By the end of the measurement period at Julian day 230, 881 TDD had accumulated in 2015 and 800 had accumulated in 2016 (Figure 5b). This was primarily due to a faster accumulation of TDD in the early season when temperatures were warmer than in 2016. After day 150, TDD increased in both years at similar rates. The mean starting date of senescence was later in 2016 than in 2015 for four (PB, SG, CF, and NN) of the six populations with only a small change in the TL plants and an earlier start date in the EC plants in 2016 (Figure 5c). In 2016, the date of maximum green tissue occurred later than in 2015 in five of the six populations, EC being the exception (Figure 5d).

## 4 DISCUSSION

### 4.1 Ecotypic differences in biomass accumulation but not growth rate

Transplanted tussocks of *E. vaginatum* growing in a common garden at Toolik Lake, Alaska showed clear differences in the length of the leaves that they produce. The trend over both measurement years was for southern populations, from sites with longer and warmer growing seasons, to grow longer leaves. This is consistent with similar previous experiments in this system, both at Toolik Lake and at other transplant gardens along the latitudinal gradient in Alaska (Fetcher & Shaver, 1990; Shaver et al., 1986; Souther et al., 2014). The prevailing hypothesis to explain these observed differences is that the milder growing conditions in southern sites have selected for more productive phenotypes which continue to be expressed even 30 years after transplanting (Souther et al., 2014). The present study tracked leaf growth of *E. vaginatum* over a growing season and helps to explain the differences between ecotypes that have been observed in previous studies. We hypothesized that southern ecotypes would have higher growth rates. However, the present study does not support this idea. Although there was high variability in the data, there were no significant differences in growth rate between ecotypes. These data therefore lead us to suggest an alternative (but not exclusive) hypothesis that the length of the growing season at the home site produces more biomass accumulation.

### 4.2 Southern ecotypes grow later into the growing season

There was a positive relationship between the maximum length of green leaves produced and both TDD and growing season length of the home site. This result was expected given that these were correlated with the higher productivity of *E. vaginatum* observed previously (Fetcher & Shaver, 1990). In both 2015 and 2016, southern ecotypes of *E. vaginatum* reached peak green biomass later in the growing season than northern ecotypes. With similar growth rates between ecotypes, the southern ecotypes reached a higher peak biomass as a result of later onset of senescence.

In both years of this common garden study, ecotypes from sites with a longer growing season senesced later in the season. This result suggests a possible selection pressure that may have shaped these ecotypic differences. The plants in the common garden came from sites along a latitudinal gradient where the length of the growing season can differ by up to 50 days. At northern sites such as Sagwon or Prudhoe Bay, the ability to grow late into the season would be selected against because temperatures would be too low for growth and there would more likely be frost and snow cover late in the season. At southern sites, such as No Name Creek or Coldfoot, the growing season is longer and plants would have a competitive advantage if they senesced later.

These results are not consistent with other studies that examined ecotypic differentiation of phenology. For example, northern populations of three herbaceous species in temperate and boreal Europe (*Lythrum salicaria*,* Solidago altissima,* and *Solidago gigantea*) flowered earlier when grown in more southern common gardens (Olsson & Ågren, 2002; Weber & Schmid, 1998). This could be the result of selection for immediate response to spring temperature increases. The opposite was observed in populations of the North American herb *Campanula americana*; populations from Florida, Georgia, and Kentucky flowered earlier than those from further north in Michigan (Kalisz & Wardle, 1994). This result was attributed to the greater abundance of biennial morphs of the species in northern states (Kalisz & Wardle, 1994). *E. vaginatum* lives much longer than the species mentioned above (Mark, Fetcher, Shaver, & Chapin, 1985), and thus, its phenology may be subject to different selection pressures.

The hypothesis that ecotypic differences in senescence timing may be controlled by an adaptation to growing season length is supported by experimental evidence from Toolik Lake. Rosa et al. (2015) showed that if the growing season is artificially brought forward by removing snow in May, the buds of seven of eight dominant tundra plant species, including *E. vaginatum*, will break earlier, but also senesce earlier. As a result, there was no increase in growing period, suggesting that annual growth time is under genetic control shaped by selection pressure (Rosa et al., 2015). Another experimental early‐season increase in snow in the high Arctic (Svalbard) produced a similar effect whereby late onset of growth results in late senescence for all species studied (Semenchuk et al., 2016). As with Rosa et al. (2015), the change in the timing of phenology had no effect on the period of active growth, suggesting a genetic control over the window for growth in arctic plants (Semenchuk et al., 2016). Consequently, the present study and previous work may have important implications for future vegetation dynamics under climate change. Potential growing seasons are getting longer in the Arctic as a result of both earlier melt of the winter snowpack and later freeze‐up (Euskirchen et al., 2006; Park et al., 2016). Growth of short‐season plants may start earlier, but under genetic constraints they will senesce earlier despite favorable growing conditions in the late season. To better understand how genetic constraints will feed into interspecific competitive interactions, common garden studies of phenology need to be performed for different tundra PFTs. We hypothesize that groups such as deciduous shrubs can extend their window of growth in response to longer growing seasons because they have shown to be phenotypically plastic (Bret‐Harte et al., 2001) in some traits, but this still needs to be tested regarding phenology.

### 4.3 Leaf growth and phenology not responsive to experimental warming

Neither leaf growth rate nor timing of senescence responded to experimental warming. This supports our hypothesis that *E. vaginatum* ecotypes have been shaped by strong selection pressures and have a very limited capacity to respond to changes in environmental conditions. A previous warming experiment at Toolik Lake observed increased leaf production of *E. vaginatum* in response to warming (using a similar method) but only in the early season, after which no difference in growth rate was observed between warmed and control plants (Sullivan & Welker, 2005). It is therefore possible that we would have seen differences between treatments if warming was applied earlier in the season (mid‐May). Other warming experiments have resulted in no increase in photosynthetic rate of *E. vaginatum* (Starr, Oberbauer, & Ahlquist, 2008), no increase in growth rate (Natali, Schuur, & Rubin, 2012), or negligible effects on vegetative growth in graminoids (Arft et al., 1999). We conclude that differences between ecotypes observed by Fetcher and Shaver (1990) are not due to differences in growth rate. Thus, no specific ecotype will have a selective advantage under climate change due to growth rate.

The finding that the growth rate of leaves of *E. vaginatum* does not respond to warming is relevant in light of a rapidly changing arctic climate and the increased dominance of likely more competitive plants (Myers‐Smith et al., 2011). There is still uncertainty surrounding the relative response of different PFTs to future climate change (Elmendorf, Henry, Hollister, Bjork, Bjorkman, et al., 2012), especially that of phenology (Oberbauer et al., 2013); however, evidence thus far would imply that deciduous shrubs that are already present in the tundra represent the most negative future competitive interaction to *E. vaginatum*. Common tundra shrubs such as *Betula nana* have been shown to exhibit developmental plasticity (e.g., increased branching, canopy density) in response to release from abiotic stress (Bret‐Harte et al., 2001). Warming experiments with open‐top chambers and greenhouses cause an increase in canopy height and cover of deciduous shrubs in the low Arctic (Elmendorf, Henry, Hollister, Bjork, Bjorkman, et al., 2012; Walker et al., 2006). Experimental warming increases the cover of graminoids as well as shrubs, but the effect is more variable and less definitive (Elmendorf, Henry, Hollister, Bjork, Bjorkman, et al., 2012). In fact, long‐term monitoring of control plots in the ITEX has also observed increases in deciduous shrub cover in comparison with sedges which have shown, on average, very little change (Elmendorf, Henry, Hollister, Bjork, Boulanger‐Lapointe, et al., 2012). With this in mind, it is becoming clearer that prominent graminoid species such as *E. vaginatum* may be at a competitive disadvantage in relation to faster‐growing species with growth strategies that can respond more quickly to more benign growing conditions (Bret‐Harte et al., 2001).

The experiments mentioned in the previous paragraph used populations of graminoids from north of the tree line. They support the hypothesis that graminoids, including *E. vaginatum*, are likely to be outcompeted by deciduous shrubs in a warming climate, although the results of competition may not become evident until 10 years or more have passed (Elmendorf, Henry, Hollister, Bjork, Boulanger‐Lapointe, et al., 2012). With the exception of the present study, no warming experiments have used graminoid populations from south of the tree line. Thus, it is difficult to predict how the populations of *E. vaginatum* from south of the tree line will respond to climate warming, although they are apparently able to maintain themselves for more than 100 years under conditions that prevailed prior to the early 1980s (Mark et al., 1985). It is possible that gene flow from the south may be able to produce a phenotype that is better able to compete with the shrubs, although it is not clear whether it will be able to keep up with the projected rate of warming (McGraw et al., 2015).

### 4.4 Phenology is more responsive to interannual climate variation than in situ warming

While there were differences in the timing of senescence between ecotypes, there was no significant effect of warming on timing of senescence, a finding that concurs with other warming studies (Bjorkman, Vellend, Frei, & Henry, 2017; Rosa et al., 2015) as well as long‐term datasets which show that phenology of arctic plants is relatively unresponsive to recent climate change (Oberbauer et al., 2013). A long‐term warming experiment also found that phenology of high arctic plants was not responsive to warming, but year‐to‐year variation in phenology could be explained by snowmelt date (Bjorkman, Elmendorf, Beamish, Vellend, & Henry, 2015). Temperatures are warming in arctic ecosystems at unprecedented rates (Serreze & Barry, 2011), but our data suggest that warming within the range of our OTC treatment (2.4°C on sunny days) may not delay the onset of senescence even if conditions are still conducive for continued photosynthesis.

In the present study, plants from four of the populations showed the first signs of senescence later in 2016 than in 2015. Similarly, the date at which maximum green leaf length was observed (which integrates across leaves on a tiller) was later for five of the populations. The difference in phenology coincides with differences in abiotic conditions between 2015 and 2016. Melt of the winter snowpack occurred at approximately the same time in both years, but after this the 2 years diverged substantially. Other work has shown that that timing of snowmelt influences phenological stages (Bjorkman et al., 2015; Rosa et al., 2015; Semenchuk et al., 2016), but on an annual basis, photoperiod and warmer temperatures are the best predictors of bud break (Rosa et al., 2015). In the present study, the snow melted at a similar time, but temperatures stayed lower later in 2016 compared to 2015. With the assumption that all the ecotypes initiated growth at the same time and that length of growing season remains consistent (Rosa et al., 2015), the later senescence and peak biomass in 2016 suggest that the growing season started later. In this case, the alleviation of cold temperatures may be the cue for growth initiation. The current understanding of the start of the growing season and subsequent phenological phases is that the timing of snowmelt is important (Bjorkman et al., 2015; Rosa et al., 2015), but temperature has a significant predictive role (Rosa et al., 2015); therefore, air temperature succeeding snowmelt merits further study.

It is notable that the clinal difference between ecotypes was reduced in 2016 and 2015. This could simply be due to interannual variation which may have a disproportionate effect on the patterns that we observed due to the low sample size in this experiment or it could reflect a number of other factors that relate to the biology of *E. vaginatum*. They could be exhibiting a slow acclimation to the new site at Toolik; however, the plants had already been in place for 4 years in 2015 and transplants continue to exhibit ecotypic differentiation up to 30 years after transplanting (Souther et al., 2014). Alternatively, the growth and senescence of tillers could be influenced by environmental conditions and metabolism from the previous year. New leaves of *E. vaginatum* are set the year prior to their growth (Shaver & Laundre, 1997), and some of the patterns that we observe in their growth may be influenced by factors such as carbohydrate storage from the previous growing season (Chapin, Shaver, & Kedrowski, 1986). It is clear that a longer record of the growth patterns of these ecotypes in a common garden will be required in order to elucidate all of the drivers of patterns that we observe.

### 4.5 Phenological cues in arctic ecosystems

The differences in the timing of the onset of senescence and maximum green date between ecotypes suggest that each has its endogenous rhythm of growth and senescence or that the ecotypes were responding differently to an environmental cue. The endogenous rhythm hypothesis is supported by phenological studies in the Arctic, which show that snowmelt timing is a predictor of senescence across a range of PFTs (Bjorkman et al., 2015; Rosa et al., 2015; Semenchuk et al., 2016). Under this hypothesis, once growth is initiated, it proceeds for a fixed number of days until senescence is initiated. For *E. vaginatum* ecotypes, this rhythm would be adaptive for variance across the latitudinal gradient from which each originates, likely based on environmental conditions. This is supported by our data which suggests senescence and maximum green date are closely linked to TDD and growing season length. However, unusual events such as a period of below normal temperatures (in 1981 at Eagle Creek, AK) can lead to premature senescence of the majority of a community (McGraw, Chester, & Stuart, 1983). Thus, while there is support for endogenous rhythms in *E. vaginatum,* there are other factors that can affect growing period.

Ecotypes are also potentially responding to environmental cues that could, in part, be driving the adaptive endogenous rhythm. As senescence and maximum green day increase with decreasing latitude of the site of origin, both photoperiod and temperature could be cues, and have been identified as consistent predictors of senescence in the arctic flora (Rosa et al., 2015). Photoperiod and light quality are closely associated and predicable on an annual basis. However, photoperiod at high latitudes is poorly defined due to the 24‐hr daylight. At Toolik Lake, the sun remains above the horizon from day of the year 140 (May 20) through day 204 (July 23), which comprises most of the growing season and includes the period when senescence of the northern populations is initiated (Figure 1). At Tromsø, Norway (69°39′N), which is only slightly farther north than Toolik Lake, photoperiod undergoes a short period of rapid lengthening in mid‐May followed by a period of no change once the sun is above the horizon for 24 hr (Nilsen, 1985). In late July, there is a corresponding period of rapid shortening followed by a period of gradual shortening (Nilsen, 1985). Because the pattern of rapid lengthening and shortening of the photoperiod varies greatly with small changes in latitude (Nilsen, 1985), it could serve as a phenological cue for plant populations native to high latitudes.

Another related cue that varies less drastically than photoperiod is light quality that could be measured by the ratio (R:FR) of red (660 nm) to far‐red (730 nm) light (Mølmann et al., 2006; Nilsen, 1985). As the sun drops below 10° above the horizon, red light is absorbed by the atmosphere and the R:FR ratio decreases. At Tromsø during the summer solstice, R:FR drops from 1.05 to 0.9 at midnight (Nilsen, 1985). Farther south at 60°N, it drops to 0.65 because the sun goes below the horizon, and farther north at 78°N, it hardly changes because the sun is above the horizon. R:FR ratio has been shown to affect the balance between the active and inactive forms of phytochrome (Leyser & Day, 2015), which is in turn responsible for germination and flowering as well as morphological changes in response to shading. Phytochrome seems to perform the same functions in the Cyperaceae as in other families. Kettenring, Gardner, and Galatowitsch (2006) found that germination of wetland *Carex* sp. (Cyperaceace) was inducible and reversible by alternating applications of red and far‐red light, which strongly suggests activity of phytochrome. Fetcher (1985) found increased tillering in response to a likely change in light quality following the removal of shrubs and mosses from tussocks of *E. vaginatum* (with no concurrent change in nutrients with treatment)*. *R:FR would also be clearly linked to the timing of the onset of winter which would be dramatically different on the far ends of the latitudinal gradient of the ecotypes; thus, R:FR should be a consistent predictor of seasonal change. We propose that as the ecotypes are from different latitudes, they differ in their sensitivity to R:FR changes. In this case, ecotypes from north of Toolik Lake (SG and PB) are more sensitive to R:FR light change and senesce earliest. Southern ecotypes would require a more pronounced R:FR reduction to initiate senescence, hence senescing later when in a common garden. If this annual rhythm is a genetic constraint, it could have repercussions for the long‐term fitness of *E. vaginatum*, especially if southern ecotypes have a limited capacity for gene flow north, where they would have a better ability to take advantage of the longer potential growing seasons.

## 5 CONCLUSIONS

The phenology of *E. vaginatum* ecotypes grown in a common garden shows that differences in biomass observed in previous transplant experiments are in part due to southern ecotypes growing later into the season, not that they have a higher growth rate than northern ecotypes. The data suggest that the difference in timing of senescence could be caused by local adaptations whereby ecotypes grow for different amounts of time, depending on the length of the growing season at their home site. The comparison of phenology over two contrasting growing seasons shows that the timing of senescence is responsive from year to year to abiotic conditions. When conditions were suitable to start growth earlier in the year, most ecotypes in this study reached their peak biomass and started to senesce earlier. Therefore, differences in phenology between ecotypes were retained over the 2 years. These results have implications for the future fitness of *E. vaginatum* with rapid climate change (IPCC 2013) along with lengthening of the growing season (Park et al., 2016). If the length of time that *E. vaginatum* grows in any given season does not vary, these locally adapted ecotypes may not be able to take advantage of longer, warmer summers if spring also starts early. In this scenario, *E. vaginatum* would suffer adaptive lag (McGraw et al., 2015), while other species such as shrubs, which may hold a competitive advantage in a warming Arctic (Elmendorf, Henry, Hollister, Bjork, Bjorkman, et al., 2012), would accelerate the community change that is already being observed in arctic landscapes (Myers‐Smith et al., 2011).

## AUTHORS’ CONTRIBUTIONS

NF, JT, and MM set up the experiment. TP and MC collected and analyzed the data. TP, NF, MM, and JT wrote the manuscript.
