# Supplementary material for: Ecotypic differences in the phenology of the tundra species Eriophorum vaginatum reflect sites of origin
Source: Ecol Evol. 2017 Oct 19;7(22):9775–86. doi: 10.1002/ece3.3445 (PMC5696421; doi:10.1002/ece3.3445)
Supplement: Supplementary file 1 [file ECE3-7-9775-s001.docx]

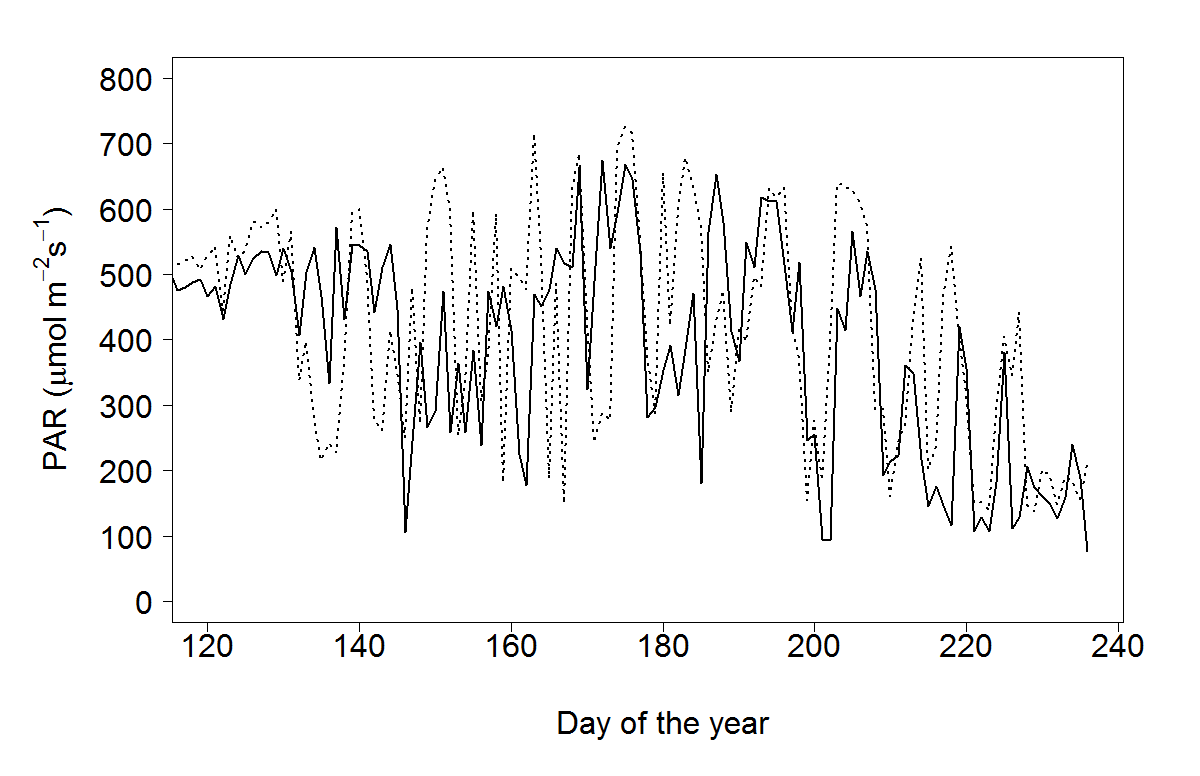


Figure S1: Photosynthetically active radiation (PAR) data at Toolik Lake Field Station over the growing season in 2015 (solid line) and 2016 (dotted line). Data are daily average values.
